# Racial and ethnic disparities in diagnostic efficacy of comprehensive genetic testing for sensorineural hearing loss

**DOI:** 10.1007/s00439-021-02338-4

**Published:** 2021-09-13

**Authors:** Michelle M. Florentine, Stephanie L. Rouse, Jihyun Stephans, David Conrad, Josephine Czechowicz, Ian R. Matthews, Anna K. Meyer, Garani S. Nadaraja, Rajan Parikh, Jordan Virbalas, Jacqueline E. Weinstein, Dylan K. Chan

**Affiliations:** 1 Department of Otolaryngology-Head and Neck Surgery, University of California-San Francisco, 2233 Post Street, Third Floor, San Francisco, CA 94115 USA; 2 Division of Pediatric Otolaryngology-Head and Neck Surgery, University of California-San Francisco, 2233 Post Street, Third Floor, San Francisco, CA 94115 USA; 3 Sackler School of Medicine, Tel Aviv University, Tel Aviv, Israel

## Abstract

Understanding racial and ethnic disparities in diagnostic rates of genetic testing is critical for health equity. We sought to understand the extent and cause of racial and ethnic disparities in diagnostic efficacy of comprehensive genetic testing (CGT) for sensorineural hearing loss (SNHL). We performed a retrospective cohort study at two tertiary children’s hospitals on a diverse cohort of 240 consecutive pediatric patients (76% publicly insured, 82% non-White) with SNHL of unknown etiology who underwent CGT. Definite and possible genetic diagnoses were assigned for each patient, representing the likelihood of a genetic cause of hearing loss. Associations between diagnostic rates were examined. 3.8 ± 2.1 variants were detected per patient; this frequency did not vary between White/Asian and Hispanic/Black cohorts. Overall, 82% of variants were variants of uncertain significance (VUS). Compared with White and Asian subjects, variants identified among Hispanic and Black children were less likely to be classified as pathogenic/likely pathogenic (15% vs. 24%, *p* < 0.001), and Hispanic and Black children were less likely to have a definite genetic diagnosis (10% vs. 37%, *p* < 0.001). The adjusted odds ratio for definite genetic diagnosis in Black and Hispanic children compared with White and Asian children was 0.19. Expanding genetic diagnostic criteria to include predicted deleterious VUSs reduced these disparities between White/Asian and Hispanic/Black children, with comparable molecular diagnostic rates (41% vs. 38%, *p* = 0.72). However, in silico predictions are insufficiently valid for clinical use. Increased inclusion of underrepresented groups in genetic hearing-loss studies to clinically validate these variants is necessary to reduce racial and ethnic disparities in diagnostic efficacy of comprehensive genetic testing.

**Supplementary Information:**

The online version contains supplementary material available at 10.1007/s00439-021-02338-4.

## Introduction

Hearing loss is the most common congenital sensory deficit, affecting one in 500 newborns (Morton and Nance 2006; Fortnum et al. 2001). 50% of bilateral sensorineural hearing loss (SNHL) is estimated to be caused by genetic factors (Marazita et al. 1993; Smith et al. 2005). Identifying an etiology for childhood SNHL can assist in prognosis and guide management in deaf and hard-of-hearing (D/HH) children (Kimberling et al. 2010; Shearer et al. 2019). Additionally, early identification of syndromic forms of SNHL, prior to the development of overt syndromic phenotypes, can significantly affect management and counseling (Brodie et al. 2020). Consensus statement from the International Pediatric Otolaryngology Group recommended comprehensive genetic testing (CGT) in etiologic testing for children with bilateral SNHL (Liming et al. 2016).

Genetic testing is valuable in the clinical management and understanding of pediatric hearing loss; however, the diagnostic rate has been reported to vary widely, from 10 to 83% (Shearer and Smith 2015). This is in part due to the discrepancy between the vast increase in the amount of genetic information readily available with the advancement and increasing availability of next-generation sequencing (NGS) and our ability to interpret the clinical significance of identified variants. With over 150 genes implicated in SNHL, testing routinely yields a large number of novel variants, most of which are single-nucleotide changes with a small number of indels and copy-number variants (Smith et al. 2005; Hilgert et al. 2009; Shearer et al. 2011). The interpretation of sequence variants is a crucial element of accurate genetic diagnosis, and discrepancies in variant interpretation can have serious implications for patient care (Amendola et al. 2016; Booth 2018; Harrison et al. 2016). The American College of Medical Genetics and Genomics (ACMG) and Association for Molecular Pathology (AMP) have published recommendations for the interpretation of sequence variants (Richards et al. 2015). These guidelines require substantial evidence to categorize a variant as disease-causing (Oza et al. 2018), and the paucity of available evidence constrains diagnostic power for populations that are historically underrepresented in genetic studies. Understanding how the efficacy and constraints of genetic testing are affected by demographic disparities is a critical concern when considering health equity.

SNHL is in many ways an ideal human genetic disease model in which to explore complexities of genetic testing—it is a common, narrowly defined, quantifiable clinical entity with a precisely delineated group of highly penetrant genes associated with a clear phenotype. Lessons learned in the study of SNHL may be applicable to the treatment of a range of genetic disorders. Racial and ethnic disparities in genetic testing in hearing loss have been observed. The rate of diagnosis as well as spectrum of genes and variants varies widely by ethnic groups, but the underlying cause of this disparity is not well described (Sloan-Heggen et al. 2016; Yan et al. 2016). The majority of studies have focused on *GJB2*, yet causative variants in these genes are mostly found in people of European and Asian descent (Chan and Chang 2014). In contrast, *GJB2* variants are rarely the cause of SNHL in Black populations (Lebeko et al. 2015).

In recent years, CGT has become more accessible due to decrease in expense and broadening of insurance coverage, increasing the opportunity to examine genetic testing outcomes in historically underrepresented populations. In this study, we report on the molecular diagnostic efficacy of CGT for SNHL in a diverse pediatric population, with a focus on examining the extent and cause of disparities in diagnostic rate and variant distribution among children from different racial and ethnic populations.

## Methods

### Study population

We performed a retrospective chart review of 240 consecutive pediatric patients with an unknown etiology of SNHL at two tertiary children's hospitals (UCSF Benioff Children’s Hospital Oakland, UCSF Benioff Children’s Hospital San Francisco) who underwent comprehensive hearing-loss gene-panel testing from 2018 to 2020. Samples were obtained by blood draw or cheek swab. Patients were not excluded based on physical exam, imaging, or other findings. This study was approved by the Institutional Review Board at UCSF.

### Demographics

Gender and insurance were extrapolated from the patients’ electronic medical record. Ethnicity, race, and primary home language were based on parents’ self-report, with children categorized as Non-Hispanic White (White), Non-Hispanic Black (Black), Non-Hispanic Asian (Asian), Hispanic, any race (Hispanic), and Other or Unknown (which included Pacific Islander, Native American, Mixed/Multi-race, or Declined to State).

### Clinical history

Clinical data were collected from otolaryngology and audiology reports. This included newborn hearing screening (NHS) results as well as earliest and most recent audiogram results. Hearing-loss onset was considered congenital if the patient referred on their NHS and post-natal if the patient passed their NHS. In cases where an NHS result was unavailable, hearing-loss onset was categorized as unknown. Baseline audiogram results are reported from the earliest audiogram report available. Hearing-loss laterality and baseline hearing-loss level were determined using pure-tone average (PTA) for thresholds between 0.5 and 4 kHz. Hearing loss was defined as follows: unilateral (PTA > 15 dB in one ear only); bilateral (PTA > 15 dB in both ears); hearing level (based on worse-ear level): normal (PTA < 15 dB HL), slight-mild (15–40 dB), moderate (41–55 dB), moderately severe (56–70 dB), severe (71–90 dB), and profound (> 90 dB). Comorbidities were defined as developmental or medical comorbid conditions that might indicate a syndromic hearing loss.

### Genetic data

Hearing-loss gene-panel testing (GeneDx) was conducted by targeted gene capture followed by massively parallel sequencing. The panel encompassed 146 nuclear genes and 6 variants in 4 mitochondrial genes accounting for non-syndromic and syndromic SNHL. Sequencing of coding regions and splice junctions with selected deletion/duplication analysis and copy-number variant detection was performed (GeneDx 2015). Each variant was classified on the clinical genetic testing report as benign (B), likely benign (LB), variant of uncertain significance (VUS), likely pathogenic (LP), or pathogenic (P) based on the ACMG 2015 Guidelines, and “definite genetic diagnosis” made based on P/LP variants and inheritance pattern (ACMG 2015; Richards et al. 2015).

To examine the distribution of variants classified based on an entirely in silico scheme, we classified VUSs as predicted benign or deleterious, based on PROVEAN (Protein Variation Effect Analyzer) score [< = − 2.5: predicted deleterious (VUS-D); > − 2.5: predicted benign (VUS-B)]. Frameshift VUSs leading to premature truncation and large deletions were classified as VUS-D. Splice-site VUSs and large duplications were classified as unknown (VUS-U). For VUSs, inheritance was defined to accord with the most common mode of inheritance for P/LP variants of that gene. In some cases where a gene exhibits both patterns, inheritance pattern was designated for the VUSs based on clinical history (Supplemental Fig. 1). Several genes with a digenic inheritance pattern were additionally identified (Supplemental Fig. 2). In cases of uncertain inheritance pattern, conflicts were resolved on discussion between the primary and senior authors (MMF, SLR, and DKC). Based on the in silico VUS predictions and these inheritance patterns, in silico “possible genetic diagnoses” were made: subjects with two VUSs in the same AR gene or a single VUS-D in an AD gene were defined as having a “possible genetic diagnosis;” for these individuals, clinically validated P/LP classification of their VUSs in the future would allow a definite genetic diagnosis to be made.

### Statistical analyses

Descriptive statistics are presented as percentages or means ± standard deviations. ANOVA was used to identify predictors of our outcomes of interest. Binomial logistic regression was used to assess for predictors and confounding variables.

## Results

### Sample population and hearing-loss gene-panel testing outcomes

**Fig. 1:**
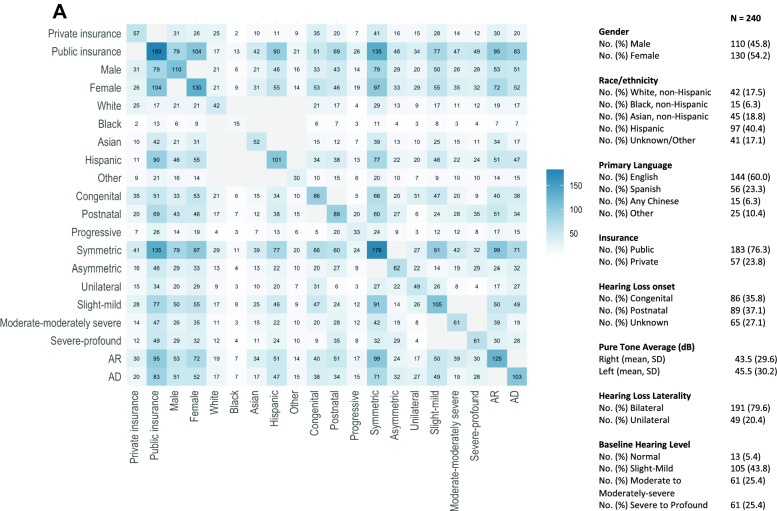
Study population demographic and clinical characteristics. **A.** Blue shading and numbers reported in individual boxes indicate the number of patients with the paired criteria delineated by row and column. **B.** Categorical variables were reported as both a number and percentage. Descriptive analysis of continuous variables is reported as a mean and standard deviation for normally distributed variables

Data were collected from 240 children, 0–22 years old, with SNHL (Fig. 1). The study population was racially, ethnically, and linguistically diverse, with a majority publicly insured (76%). 36% had congenital hearing loss. Children represented a wide range of hearing levels and were predominantly bilaterally affected. Universal public insurance access made possible equitable utilization of hearing-loss gene-panel testing, such that children in different racial and ethnic groups had comparable clinical characteristics; specifically, hearing-loss laterality, hearing level, and comorbidities were not statistically significantly different across Asian, Black, Hispanic, and White subjects. White subjects were more likely to have congenital hearing loss (Table 1).

**Table 1:** Comparison of clinical characteristics across racial and ethnic groups with reported *p* value of one-way ANOVA

	White	Black	Asian	Hispanic	Other/unknown	*p* value
% Bilateral	78.6	80.0	80.8	80.2	76.7	0.99
% Severe/profound	28.6	26.7	21.2	23.8	33.3	0.40
% Congenital	50.0	40.0	28.9	33.7	33.3	< 0.001
% With comorbidities	23.8	26.7	5.8	19.8	20.0	0.12

All 240 children underwent comprehensive hearing-loss gene-panel testing. 944 variants were identified in our study population (Supplemental Tables 1 and 2), with an average of 3.8 ± 2.1 variants identified per patient. The majority of variants were Variants of Uncertain Significance (VUSs; 82%). Of the remainder, 14% were identified as Pathogenic (P) and 5% Likely Pathogenic (LP); we analyzed these together as P/LP variants. 22% of subjects overall received a definite genetic diagnosis based on the presence of P/LP variants with the appropriate inheritance pattern.

### Racial and ethnic disparities in genetic testing outcomes

**Fig. 2:**
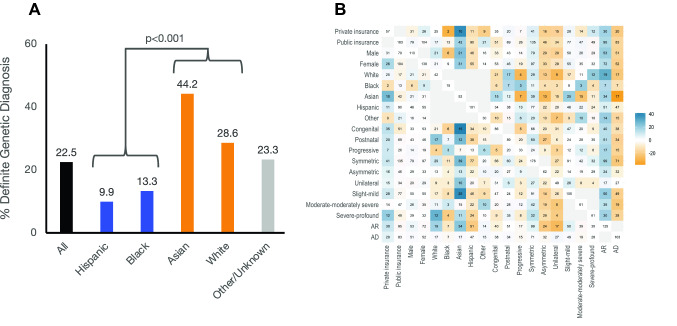
Definite genetic diagnostic rate. **A.** Distribution of definite genetic diagnoses (Patient MD = 4) across race/ethnicity groups. Definite genetic diagnostic rate was compared by ANOVA between dichotomized race/ethnicity groups (White/Asian and Black/Hispanic). **B.** Distribution of definite genetic diagnosis across insurance, sex, racial-ethnic group, onset, severity, laterality, and inheritance characteristics. Coloring/shading is indicative of difference of diagnostic rate from the average diagnostic rate adjusted by row: orange indicates below the average diagnostic rate for patients with the characteristic defined by the row, white indicates a diagnostic rate average for patients with the characteristic defined by the row, and blue indicates higher diagnostic rate for patients with the characteristic defined by the row. Numbers in boxes indicate the number of patients who received a definite genetic diagnosis, defined as Patient MD of 4, with paired characteristics of the column and row

We sought to assess for differences in definite genetic diagnostic rates across racial and ethnic groups. We found that Asian and White children had a higher rate of definite genetic diagnoses (26% and 46%, respectively) when compared to Black and Hispanic children (10% and 13%, respectively) (Fig. 2). The majority of these diagnoses (17/24 Asian, 6/10 Hispanic, and 5/11 White) were attributable to variants in *GJB2*, particularly the c.109G>A and c.35delG variants common in Asian and European populations, respectively**.** However, even when these variants are excluded, the disparity remains, with diagnostic rates still lower in Black and Hispanic subjects (7% for these groups together, compared to 20% of White and Asian subjects).

To reflect this disparity for subsequent analyses, we dichotomized race and ethnicity into a group of Black and Hispanic children and a group of Asian and White children. This grouping is further justified by our finding that published literature on hearing-loss genetics underrepresents African and Latino American subjects by 20–30-fold compared with Asian and European subjects (Rouse et al. 2021). Thus, Black and Hispanic children in our study constitute an underrepresented minority (URM) group in hearing-loss genetics compared with control (Asian and White) subjects. On one-way ANOVA, there was a significant association between definite genetic diagnosis and race/ethnicity, with only 10% of URM children receiving a definite molecular diagnosis compared with 37% of White and Asian controls (*p* < 0.001).

**Fig. 3:**
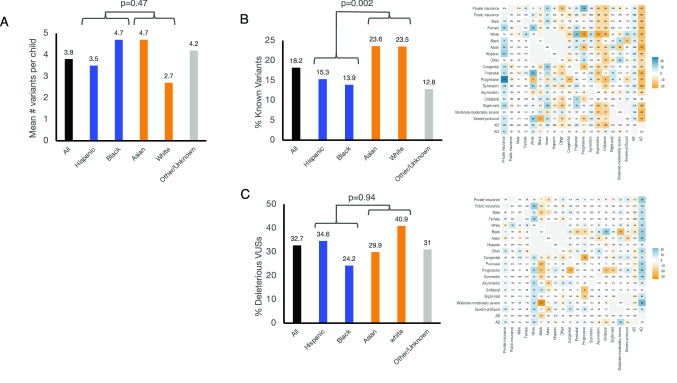
Variant distribution. **A.** Mean number of variants identified per child across race/ethnicity groups with reported p-value of ANOVA for mean number of variants vs dichotomized race/ethnicity (White/Asian and Black/Hispanic). **B.** Known Variant rate. Left: Distribution of Known Variants across race/ethnicity groups. Known Variant rate was compared by ANOVA between dichotomized race/ethnicity groups (White/Asian and Black/Hispanic). Right: Distribution of Known Variants across characteristics is shown. Color scheme is as described in Fig 2. **C.** Predicted deleterious VUS rate. Left: Distribution of predicted deleterious VUSs (by PROVEAN prediction) across race/ethnicity groups. Deleterious VUS rate was compared by ANOVA between dichotomized race/ethnicity groups (White/Asian and Black/Hispanic). Right: Distribution of deleterious VUSs across characteristics is shown. Color scheme is as described in Fig 2

We sought to understand why the definite genetic diagnostic rate varies by race and ethnicity. One possible contributor to this disparity is a difference in the number of rare variants identified between the two groups. After NGS, rare variants are identified by filtering based on racial/ethnic-group-specific allele frequency. One-way ANOVA demonstrated no significant difference between URM and control groups in the number of variants identified per patient (3.8 vs 3.6 variants, *p* = 0.47; Fig. 3A). Therefore, the number of rare variants detected was unlikely to contribute to the association between race/ethnicity and definite genetic diagnostic rate.

We then probed the hypothesis that the variants identified in our control population were better studied than those found in URM subjects and therefore were more likely to lead to a genetic diagnosis. To assess this, we examined the percentage of P/LP variants, many of which are known to be pathogenic because they have been previously described or published. Overall, only 18% of the variants were P/LP variants, with the remainder (82%) classified as VUSs. One-way ANOVA demonstrated that P/LP variants were significantly less common in URM compared with control children (15.0% vs 24.0%, *p* = 0.002; Fig. 3B).

This disparity in P/LP variants suggests that the body of knowledge contributing to variant classification is inequitable with respect to race and ethnicity. Alternatively, URM children may simply have a lower proportion of deleterious variants in hearing-loss genes. To probe this further, we analyzed variants that were categorized as VUSs and segregated them based on PROVEAN prediction. Because the PROVEAN predictions are based on the in silico predicted effect of the genetic variant on protein function, they should be independent of prior literature, and therefore less subject to prior unequal representation of racial and ethnic groups. We dichotomized VUSs into predicted deleterious VUSs and predicted benign or unknown VUSs, and found no significant difference between the URM and control groups (*p* = 0.78; Fig. 3C).

**Fig. 4:**
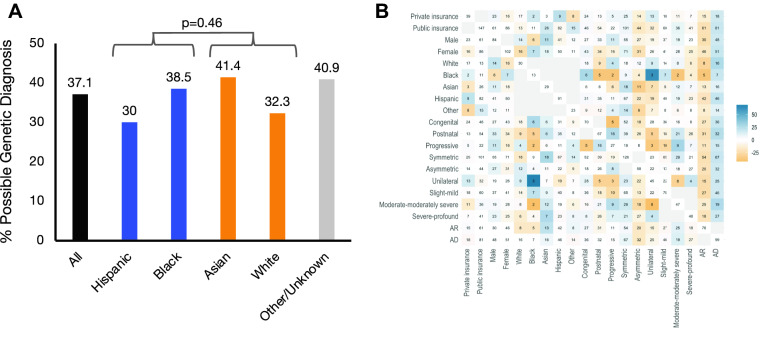
Possible genetic diagnostic rate. **A.** Distribution of possible genetic diagnoses (Patient MD = 3) across race/ethnicity groups. Possible genetic diagnostic rate was compared by ANOVA between dichotomized race/ethnicity groups (White/Asian and Black/Hispanic). **B.** Distribution of possible genetic diagnosis across characteristics is shown. Color scheme is as described in Fig. 2

We tested the impact of this racial and ethnically agnostic in silico method of variant prediction on genetic diagnostic rate. Subjects with a single predicted deleterious VUS in an autosomal dominant gene or two VUSs in an autosomal recessive gene were defined as having a “possible genetic diagnosis” based on the supposition that clinical validation of these VUSs as P/LP would yield a genetic diagnosis. Using this in silico classification scheme, URM and control children had comparable possible genetic diagnostic rates (41% vs 38%, *p* = 0.72; Fig. 4). Thus, when an in silico, racial, and ethnically agnostic variant classification scheme was used, the racial and ethnic disparity in genetic diagnostic rate was eliminated.

The comparison between genetic testing outcomes and clinical characteristics in URM and control groups is summarized in Table 2. Even though the two groups were indistinguishable clinically and had a comparable number of rare variants detected, URM subjects had significantly fewer P/LP variants, more VUSs, and worse definite genetic diagnostic rate. To determine the effect of racial/ethnic representation on genetic diagnosis adjusting for clinical and demographic confounders, we performed binomial logistic regression (Table 3). The odds of a URM (Black or Hispanic) child receiving a definite genetic diagnosis is 0.19 that of a control (White or Asian) child (95% CI 0.09–0.41, *p* < 0.001). When the diagnosis is based on race-and-ethnicity agnostic PROVEAN prediction, race and ethnicity no longer affect the overall distribution of genetic diagnoses (*p* = 0.52).

**Table 2:** Comparison of genetic outcomes and clinical characteristics between URM (Black and Hispanic) and control (Asian and White) groups

	White/Asian	Black/Hispanic	*p* value
Number of variants per child (mean, SD)	3.8 (2.1)	3.6 (2.1)	0.47
% P/LP variants	23.6	15.1	0.002
VUS (%)	76.4	84.9	< 0.001
% of VUSs that are predicted deleterious	38.7	38.1	0.78
Definite genetic diagnosis (%)	37.1	10.3	< 0.001
Possible genetic diagnosis (%)	41	38.1	0.72
% Bilateral	79.6	80.1	1.00
% Severe/profound	23.7	24.5	0.55
% Congenital	37.3	31.6	0.60
% With comorbidities	15.5	20.5	0.34
% Publicly insured	68.1	88.4	< 0.001
% Non-English-speaking	42.0	46.2	0.26

*p* values from one-way ANOVA

**Table 3:** Logistic regression analysis of definite and possible genetic diagnosis with clinical and demographic covariates, including URM status (URM (Black or Hispanic) vs. control (Asian or White), primary home language, hearing-loss laterality, hearing-loss onset, baseline hearing-loss level, and presence of comorbidities

	Definite genetic diagnosis		
	Odds ratio	*p* value	95% CI
Race/ethnicity (URM re: control)	0.19	** < 0.001**	(0.09–0.41)
Language (non-English re: English)	0.62	0.25	(0.27–1.40)
Laterality (unilateral re: bilateral)	0.28	**0.03**	(0.08–0.89)
Onset (post-natal re: congenital)	1.67	0.26	(0.68–1.50)
Hearing level (severe–profound re: mild–moderate)	0.63	0.30	(0.26–1.50)
Comorbidities (present re: absent)	0.31	**0.05**	(0.10–1.00)

	Possible genetic diagnosis		
	Odds ratio	*p* value	95% CI
Race/ethnicity (URM re: control)	0.86	0.66	(0.43–1.71)
Language (non-English re: English)	1.17	0.67	(0.58–2.37)
Laterality (unilateral re: bilateral)	0.52	0.13	(0.23–1.20)
Onset (post-natal re: congenital)	0.75	0.48	(0.34–1.67)
Hearing level (severe–profound re: mild–moderate)	0.58	0.19	(0.26–1.31)
Comorbidities (present re: absent)	0.83	0.64	(0.37–1.83)

The reference level “re:” is indicated for each variable. Results include the odds ratio, 95% confidence interval, and *p* values

Bold values indicate statistically significant associations (p < 0.05)

## Discussion

Identifying genetic etiology of hearing loss can improve clinical care. With the advent of NGS, genetic testing is readily available and has become a recommended test for etiologic workup of bilateral SNHL (Shearer et al. 2019), but this approach has its limitations. Access to CGT is often limited by economic factors, and variants are difficult to interpret. The development of massively parallel sequencing technologies has made genetic data more readily available than ever before, shifting the bottleneck in identifying molecular etiology from acquisition of data to meaningful variant interpretation that is accurate, disease-specific, and equitably representative.

We present here an analysis of the clinical efficacy of CGT in a diverse pediatric population. From 240 pediatric patients with SNHL of unknown etiology, 944 variants were identified in 132 genes. Overall, 22% of patients were diagnosed by CGT, with significant variability seen across racial and ethnic groups. Asian (46%) and White (26%) groups had significantly higher diagnostic rates than Black (13%) and Hispanic (10%) children. Though diagnostic rates were overall lower than previous reports, the trend in differences in between racial and ethnic group rates was similar (Sloan-Heggen et al. 2016). Our finding that URM subjects (Blacks and Hispanics) were less likely to receive a definite genetic diagnosis than controls (Asians and Whites) was neither attributable to clinical covariates nor the number of variants identified per child.

Instead, we found that the disparity was related to a significantly lower rate of P/LP variants (and, conversely, higher rate of VUSs) among URM subjects. This inequity in variant classification is likely due to a higher rate of prior genetic studies performed on White and Asian populations compared to Black and Hispanic ones, an inequity that has been demonstrated repeatedly (Edwards et al. 2020). Specific to hearing loss, most SNHL genes have been identified in families from consanguinity belts in the Middle East and India (Bademci et al. 2016). On the other hand, few studies have been done in people of African or indigenous American descent, due to a combination of decreased access to care as well as cultural differences, historic stigmatization, and discrimination that have contributed to avoidance of genetic testing and research (Hall and Olopade 2005, 2006).

We tested to see whether using a method of variant analysis that is agnostic to prior history of testing in different racial and ethnic groups—in silico prediction of deleterious versus benign VUSs—would eliminate this disparity in genetic diagnostic rate. Indeed, these in silico predictions were equitable between URM and control groups, and when we compared the “possible genetic diagnosis” rate, based on the in silico variant classification, URM and control subjects had comparable diagnostic rates. These findings highlight the gap in understanding of variants across these populations and the critical need for increased inclusion of underrepresented groups in genetic hearing-loss studies. Accumulating genetic data on a more diverse group of individuals with and without hearing loss will allow us to better classify VUSs as pathogenic or benign, decreasing the rate of false positives and negatives and increasing the CGT diagnostic value.

Such a classification scheme based on in silico predictions alone without additional clinical validation is clearly not sufficient to make actionable genetic diagnoses. This need for significant evidence to classify variants, however, skews definitive classifications toward populations that are better represented in the literature and variant databases (Gerhard et al. 2018; Manrai et al. 2016). Thus, the clinical value of genetic testing is higher among these groups, exacerbating disparities in treatment and understanding of disease. This is well documented in the study of genetic testing in breast cancer, cardiomyopathy, and chronic kidney disorders, in which racial and ethnic health disparities persist despite the rapid increase in genetic information (Shan et al. 2012; Landry and Rehm 2018; Gasmelseed et al. 2004).

Our study is unique in that 76% of our study population is publicly insured. While Black and Hispanic patients are less likely to have private insurance coverage and therefore less access to genetic testing, access to genetic testing was not a barrier for the Black and Hispanic patients in our study. Additionally, the clinical indications for testing and clinical features themselves were comparable across all groups. The only exception was that White subjects were more likely to have congenital hearing loss (Table 1); however, multiple regression analysis demonstrated that onset of hearing loss (congenital vs post-natal) was not significantly associated with diagnostic rate, whereas race/ethnicity was (Table 3). Because access to testing and clinical indications for obtaining testing was consistent across racial and ethnic groups, differences in diagnostic rate are likely reflective of the disparity in P/LP variants, rather than differences in patients’ clinical features.

There are several limitations to our study. Family history was not available for the majority of patients, so designation of inheritance was not based on family pedigree but instead on clinical report or OMIM classification of affected genes. Thus, the VUS reclassification we designed and tested may disproportionately inflate the role of dominant variants. We mitigated this effect by defaulting to a recessive pattern when inheritance was in question for a VUS. Due to limited family history and parental testing, we cannot confirm biallelic (in trans) variants for most subjects. Pre-clinical syndromic associations may have been missed in clinical evaluations. Though we found equivalent numbers of variants and rate of deleterious VUSs between URM and control groups, individual racial/ethnic groups did show some differences in these rates that did not match the URM/control dichotomization (Fig. 3A, C). Further study is required to understand why these differences may have occurred.

Finally, while our cohort includes many patients underrepresented in studies of genetic HL, only 15 of 240 of our patients were Black, showing the limits to inclusivity and representation even in this relatively large study. Overall, the categorization of race and ethnicity in studies of genetics is complex*.* Health disparities between vulnerable social groups such as racial and ethnic minorities are frequently based in nonbiologic characteristics such as socio-economic status. Race and ethnicity are sociocultural constructs and are treated as such in this study. It is important to consider disparities within the context of these socially defined categories, as these are the same classifications that lead to disparate treatment. As such, race and ethnicity were self-reported in our study. However, this classification can lead to imprecise designations and limitations in interpretation. Genetic ancestral analysis can provide more precision into classifying the ancestral background of individuals in genetic studies. However, conflating genetic ancestry determined by biological markers and the social construct of race and ethnicity is problematic. Understanding the difference between these constructs is critical in reporting and interpreting genetic studies.

We found that current CGT for SNHL is five times less effective in Black and Hispanic children compared to White and Asian ones, accounting for covariates (Table 3). This decreased hit rate may lead clinical providers to utilize this resource less often in these populations. Compared to White children, Black children with SNHL in the US are already half as likely to receive genetic testing (Qian et al. 2021). If applications of new technology, such as CGT, continue to be utilized disproportionally, the models generated from newly available data risk perpetuating and exacerbating health disparities (Smith et al. 2016). This has been seen in the genetic diagnosis of cancers, in which disparities in genetic testing of cancer predisposition have increased disparities in clinical management (Cragun et al. 2017; Thompson et al. 2003). Similarly, misclassification of hypertrophic cardiomyopathy has been demonstrated in African Americans due to lack of accessible genetic data from appropriate control populations (Gerhard et al. 2018).

The gap in diagnostic utility between racial and ethnic groups highlights the need for expansion of genetic knowledge among traditionally underrepresented groups of D/HH individuals. Targeted studies of underrepresented groups to understand these hearing-loss genes and variants, as well as acquisition of large-scale sequencing data from diverse populations, are necessary to close this gap. Expanding the scope of testing to involve whole-exome sequencing will allow better identification of novel variants in genes not currently represented in existing panels. While CGT is one of the strongest tools in the clinical evaluation of HL, there is a need to establish that it is equivalently useful and clinically valid across all populations, or else it threatens to exacerbate existing disparities (Smith et al. 2016). As efforts increase to develop gene therapy for hearing loss, ensuring an inclusive basis of genetic diagnosis is critical to avoid propagation of historical inequities from testing to treatment.

## Supplementary Information

Below is the link to the electronic supplementary material.

Supplementary file1 (DOCX 13 KB)

Supplementary file2 (XLSX 102 KB)

Supplementary file3 (PDF 111 KB)

Supplementary file4 (PDF 87 KB)

## Data Availability

All relevant data are included in this manuscript.
