# Supplementary material for: Racial and ethnic disparities in diagnostic efficacy of comprehensive genetic testing for sensorineural hearing loss
Source: Hum Genet. 2021 Sep 13;141(3-4):495–504. doi: 10.1007/s00439-021-02338-4 (PMC9035005; doi:10.1007/s00439-021-02338-4)
Supplement: Supplementary file 1 — Supplementary file1 (DOCX 13 KB) [file 439_2021_2338_MOESM1_ESM.docx]

**Supplemental Data Legends**

**Supplemental Figure 1. Inheritance classification.** Algorithm for inheritance pattern determination for hearing-loss genes. AD: Autosomal dominant; AR: Autosomal recessive.

**Supplemental Figure 2. Complex Inheritance classification.** Algorithm for inheritance pattern for non-simple autosomal recessive/dominant genes. AD: Autosomal dominant; AR: Autosomal recessive.

**Supplemental Table 1. List of reported variants**. All variants included in clinical reports from hearing-loss gene panel testing (GeneDx) for all subjects in this study, together with subject-level demographic and clinical characteristics. VUS: Variant of Uncertain Significance; AD: Autosomal dominant; AS: Autosomal recessive; XR: X-linked Recessive.
