# Supplementary material for: Racial and ethnic disparities in diagnostic efficacy of comprehensive genetic testing for sensorineural hearing loss
Source: Hum Genet. 2021 Sep 13;141(3-4):495–504. doi: 10.1007/s00439-021-02338-4 (PMC9035005; doi:10.1007/s00439-021-02338-4)
Supplement: Supplementary file 3 — Supplementary file3 (PDF 111 KB) [file 439_2021_2338_MOESM3_ESM.pdf]

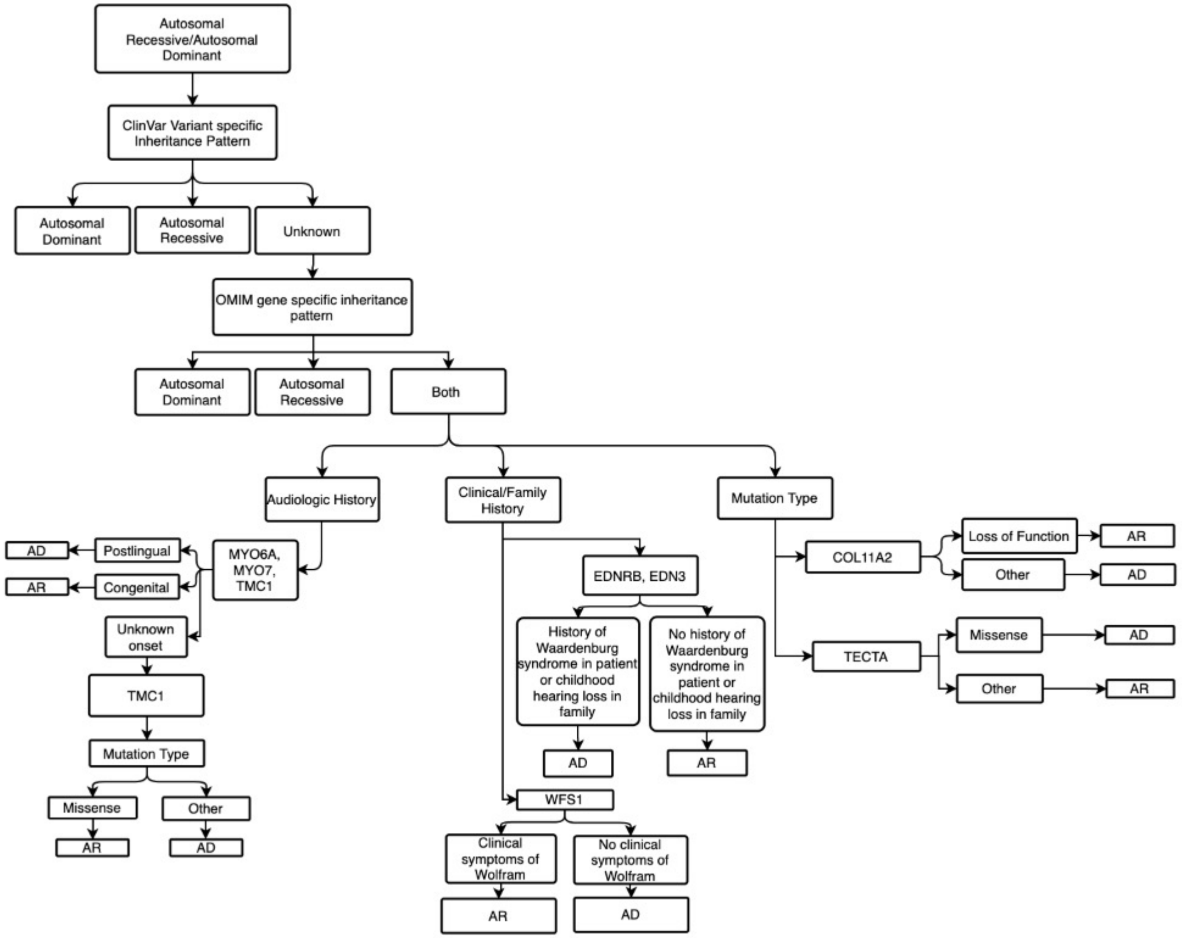

Supplemental Figure 1. Inheritance classification. Algorithm for inheritance pattern determination for hearing-loss genes. AD: Autosomal dominant; AR: Autosomal recessive.
