# Supplementary material for: Racial and ethnic disparities in diagnostic efficacy of comprehensive genetic testing for sensorineural hearing loss
Source: Hum Genet. 2021 Sep 13;141(3-4):495–504. doi: 10.1007/s00439-021-02338-4 (PMC9035005; doi:10.1007/s00439-021-02338-4)
Supplement: Supplementary file 4 — Supplementary file4 (PDF 87 KB) [file 439_2021_2338_MOESM4_ESM.pdf]

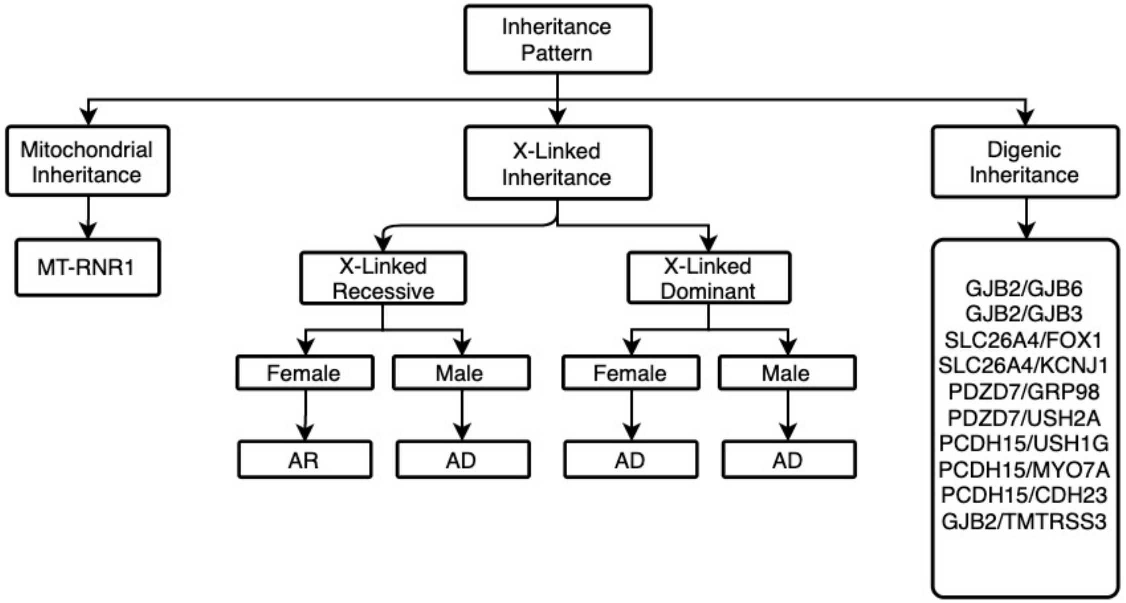

Supplemental Figure 2. Complex Inheritance classification. Algorithm for inheritance pattern for non-simple autosomal recessive/dominant genes. AD: Autosomal dominant; AR: Autosomal recessive.
